# Green synthesis of ZnO nanoparticles using *Citrus sinensis* peel: optimizing volume ratios for enhanced antibacterial and banana preservation activities

**DOI:** 10.1186/s12896-026-01145-x

**Published:** 2026-03-30

**Authors:** Alebachew Molla, Hana Bizualem, Enyew Tilahun, Tesfahun Dagnaw, Gedif Meseret, Dawit Leja, Melese Tadese

**Affiliations:** 1 Department of Biotechnology, College of Natural and Computational Science, Debre Markos University, Debre Markos, Ethiopia; 2 Department of Applied Chemistry, Adama Science and Technology University, Adama, Ethiopia; 3 Department of Chemistry, College of Natural and Computational Science, Wolaita Sodo University, Sodo, Ethiopia; 4 Department of Biology, College of Natural and Computational Science, Wolaita Sodo University, Sodo, Ethiopia; 5 Department of Biotechnology, College of Natural and Computational Science, Wolaita Sodo University, Sodo, Ethiopia

**Keywords:** Nano-biotechnology, Preservative activity, Antibacterial activity, Banana, *C. sinensis*, ZnO Nanoparticles, Green Synthesis, Chitosan Coating

## Abstract

This study explores the green synthesis of zinc oxide nanoparticles (ZnO NPs) using aqueous extract of *Citrus sinensis* peel. *C. sinensis* peel is bioactive, phytochemical rich and readily available agro-waste. The aim of this study was to investigate the effects of varying the volume ratio of zinc nitrate precursor to peel extract (1:1, 1:2, and 2:1) on the physicochemical properties, antibacterial activity, and preservative efficacy of biosynthesized ZnO NPs. Structural analysis by X-ray diffraction (XRD) confirmed the formation of crystalline hexagonal wurtzite ZnO NPs across all synthesis ratios. The 2:1 ratio exhibiting superior crystallinity and larger crystallite size (24.2 nm). Fourier transform infrared (FTIR) spectra identified zinc-oxygen bonds and confirmed the role of peel-derived biomolecules in NP capping and stabilization. Ultraviolet-visible (UV-vis) spectroscopy revealed strong absorbance peaks at approximately 370 nm, characteristic of ZnO NPs, with the 2:1 sample exhibiting the highest optical absorption. Scanning electron microscopy coupled with energy dispersive spectroscopy (SEM-EDS) and transmission electron microscopy (TEM) revealed morphological transitions from aggregated particles to elongated rod-like structures with increasing zinc nitrate ratio, confirming elemental composition of zinc and oxygen. Antibacterial activity testing using disc diffusion methods against Gram-positive and Gram-negative bacteria revealed concentration-dependent inhibition, with ZnO synthesized at a 2:1 ratio providing the most potent antibacterial activity. Furthermore, chitosan-based nano-coating incorporated with ZnO NPs significantly reduced weight loss in banana fruit preservation trials, indicating enhanced shelf life. The 2:1 ratio coating is showing the best preservation performance. This study underscores the critical role of precursor ratio in tailoring NP size, morphology, crystallinity, and bioactivity. The findings demonstrate that green-synthesized ZnO NPs using *C. sinensis* peel extract represent promising, sustainable nanomaterials for antibacterial applications and food preservation, with minimized environmental impact.

## Introduction

Nano-biotechnology is an innovative and emerging research area aiming to manufacture novel and biocompatible materials at the nanometer scale (1–100 nm) [1, 2, 3]. Compared with materials that have an undefined particle size, nanomaterials consist of small particles with a large surface area, resulting in materials that display unexpected surface area, volume, quantum size, and macro tunneling effects responsible for specific applications [4, 5]. The swift progress of nanotechnology has generated considerable interest in the synthesis and applications of nanomaterials. Among various metal oxide NPs, zinc oxide nanoparticles (ZnO NPs) have garnered considerable attention. This is due to their remarkable antibacterial, antifungal, and preservative properties [6, 7].

Traditional physical and chemical methods used to synthesize ZnO NPs often involve toxic chemicals and high-energy consumption, leading to rising environmental safety concerns [8, 9]. To mitigate these issues, green synthesis methods using biological materials have emerged as sustainable, ecofriendly, and cost-effective alternatives [10]. Plant extracts are rich in natural bioactive compounds, such as flavonoids, alkaloids, terpenoids, tannins, phenolic, and polyphenols, which can act as reducing and stabilizing agents during the synthesis of NPs [11, 12].

*C. sinensis* (sweet orange) peels are a common agro-waste product that contains abundant phytochemicals with potent antioxidant and antimicrobial properties [13, 14]. Utilizing *C. sinensis* peel extract for the green synthesis of ZnO NPs not only promotes waste valorization but also enhances the biocompatibility and functional efficiency of the NPs [15, 16].

Despite the established use of plant extracts in NP synthesis, the influence of synthesis parameters particularly the volume ratio of peel extract to zinc precursor solution on the size, morphology, and bioactivity of ZnO NPs has not been thoroughly investigated [15, 17, 18, 19, 20]. Gaining a deeper understanding of how these synthesis conditions modulate ZnO NP characteristics is essential for optimizing their effectiveness in various applications, ensuring enhanced bioactivity and improved performance as antibacterial and preservative agents [17].

ZnO NPs are widely studied for their ability to inhibit the growth of various pathogenic bacteria and fungi [21, 22, 23]. These properties make them beneficial in wound healing, antimicrobial coating, and medical device fabrication [24, 25, 26]. Their bactericidal action is often attributed to mechanisms such as the generation of reactive oxygen species (ROS), disruption of microbial cell membranes, and release of zinc ions [27, 28]. ZnO NPs also contribute to microbial cell death without significant toxicity to human cells at optimum concentration [29, 30].

The exceptional antibacterial, antifungal, and preservative properties of ZnO NPs make them highly valuable in advancing both biomedical techniques and food preservation techniques, reflecting the broader potential impact of Nano-biotechnology in the health and food safety sector [7, 31]. ZnO NPs can serve as effective preservatives due to their ability to hinder microbial spoilage, thereby extending the shelf life of perishable food products [6, 32]. Their incorporation into packaging materials and coatings can provide a protective barrier against contamination, maintaining food safety and quality [33, 34].

This study aims to biosynthesize ZnO NPs using *C. sinensis* peel extract. Additionally, it investigates how varying the volume ratio of the extract affects the physicochemical characteristics of ZnO NPs and their antibacterial and preservative activities. We hypothesize that higher zinc precursor ratios enhance crystallinity and bioactivity. The finding of this study can contribute to the development of sustainable nanomaterials with potential applications in food safety and biomedical fields.

## Materials and methods

### Material and reagents

Zinc nitrate hexahydrate [Zn(NO_3_)_2_.6H_2_O] and Sodium hydroxide (NaOH) pellets were procured from Thermo Scientifi Chemicals (USA). Ethanol was obtained from Merck (Germany). Microbiological culture media were purchased from HiMedia Laboratories (India). All chemicals and reagents used in this study were of analytical grade without further purification.

### Preparation of plant extract

*C. sinensis* fruits were purchased from local suppliers and transported to the Biotechnology Laboratory at Wolaita Sodo University. The fruits were washed several times with tap water, followed by deionized water. The peels were separated, spread out in a dark room, and dried at ambient temperature until a constant weight was achieved. The dried *C. sinensis* peels were ground into a fine powder using a grinding machine. To prepare the aqueous extract, 60 g of peel powder was added to 800 ml of deionized water and subjected to magnetic stirring and boiling at approximately 100 °C for 60 min. The extract was then cooled, filtered, and stored at 4 °C in amber bottles until further analysis [35].

### Biosynthesis of ZnO NPs

**Fig. 1:**
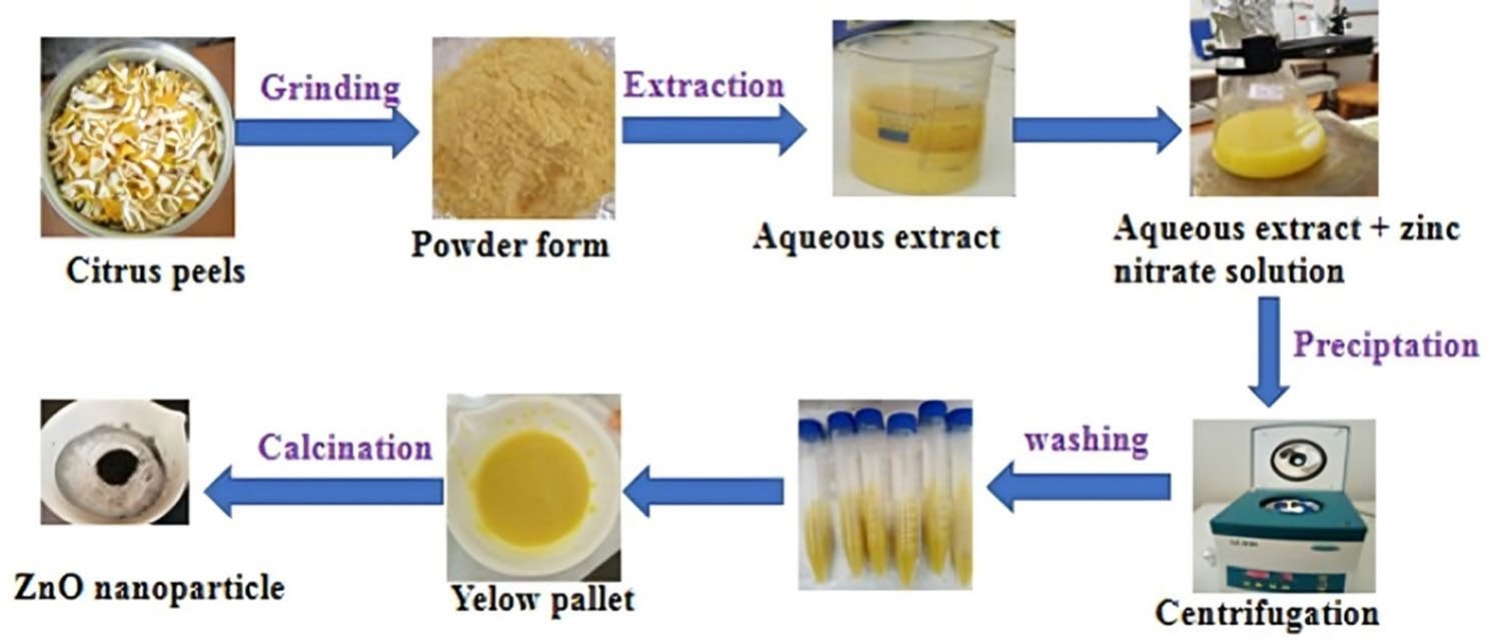
Schematic representation of the green synthesis of ZnO NPs using *C. sinensis* peel aqueous extract

ZnO NPs were synthesized by using 0.5 M of the salt precursor zinc nitrate and *C. sinensis* peel extract of different volume ratios as 1:1 (50 ml salt precursor: 50 ml peel extract), 1:2 (33.3 ml salt precursor: 66.70 ml peel extract), and 2:1 (66.70 ml salt precursor: 33.3 ml peel extract) (Fig. 1). The prepared solutions (salt precursor and peel extract) were labeled as ZnO 1:1, ZnO 1:2, and ZnO 2:1. All solutions of different volume ratios were stirred for about 4 h without heating. The pH of each of the formed suspensions was adjusted to pH 12 using 0.1 M of sodium hydroxide (NaOH) solution as a precipitating agent and stirred for 30 min to create precipitation [36]. The solution with a precipitate was centrifuged at 1500 rpm and washed four times with distilled water and absolute ethanol, dried overnight in an oven at 70 °C, and calcined at 500 °C for about 4 h under a Furness machine at 450 °C [37].

### Characterization of ZnO NPs

The biosynthesized ZnO NPs were comprehensively characterized using various analytical techniques to confirm their structural, morphological, chemical, and optical properties.

The crystalline structure and phase purity of the synthesized ZnO NPs were analyzed using X-ray diffraction (XRD). Diffraction patterns were recorded over a 2θ range of 20–80° at a scanning rate of 2°/min. The observed diffraction peaks were indexed to the hexagonal wurtzite structure of ZnO and the average crystallite size was calculated using the Debye-Scherrer quastion. Surface functional groups and biomolecules responsible for capping and stabilization were identified using FTIR spectroscopy. Spectra were recorded in the wavenumber range of 4000 –400 cm^− 1^ using the KBr pellet method. The presence of phytochemicals from *C. sinensis* peel extract and the characteristic Zn-O stretching vibration were confirmed [38].

Optical properties were investigated using a UV-Vis spectrophotometer in the wavelengeth range of 200–800 nm. The absorption spectrum revealed the characteristic excitonic peak of ZnO, and the optical bandgap energy was estimated using the Tauc plot method. Surface morphology and particle distribution were examined using field emission scanning electron microscopy operated at an accelerating voltage of 5–15 kV. Elemental composition was determined using an energy dispersive X-ray spectroscopy (EDS) detector attached to the SEM, confirming the presence of zinc and oxygen without detectable impurities. Transmission Electron Microscopy (TEM) provided further detailed visualization of the NPs, revealing their shape, size distribution, and crystallinity on the nanoscale [39].

### Antibacterial activity of ZnO NPs

The antibacterial activity of biosynthesized ZnO NPs from *C. sinensis* peel extract was tested against both Gram-positive (*Staphylococcus aureus* (ATCC25923), *Streptococcus pyogenes* (ATCC19615)) and Gram-negative bacteria (*Escherichia coli* (ATCC25922), *P. aeruginosa* (ATCC27853)) using the disc diffusion assay method [40, 41, 42]. To determine the minimum inhibitory concentration (MIC), serial concentrations of 50, 75, and 100 µg/ml were tested for each ratio (1:1, 1:2, and 2:1) of biosynthesized ZnO NPs. Ampicillin was used as a positive control. The plates were incubated at 37 °C for 24 h and examined for zones of inhibition, appearing as clear areas around the discs. The diameter of the clear zone was measured in mm using the ruler scale.

### Preservative activities of biosynthesized ZnO NPs

To evaluate the preservative activity of biosynthesized ZnO NPs, nano-coating solutions of varying concentrations were prepared in an edible chitosan solution. First, 4 g of chitosan was added to 400 ml of boiled water, stirred until all chitosan was dissolved, and cooled at room temperature. ZnO nano-coating solutions were prepared by adding 0.50, 0.75, and 1.00 g of ZnO NPs to 100 ml of chitosan solution in 500 ml conical flasks to obtain final ZnO concentrations of 0.50, 0.75, and 1.00% (w/v), respectively. 1.5% (v/v) glycerol was added to each nano-coating solution used as a plasticizer [43]. The solutions were stirred for 15 min at ambient temperature. Deionized water was used as the negative control. Healthy, pre-weighed banana fruits were soaked in the nano-coating solutions for about 1 min and then allowed to dry at room temperature for 30 min [44]. The coated bananas were placed on aluminum foil-covered benches in a dark room, and weight loss was recorded at 1 day’s intervals for 14 days.

## Result and discussion

### X-ray diffraction (XRD) analysis

**Fig. 2:**
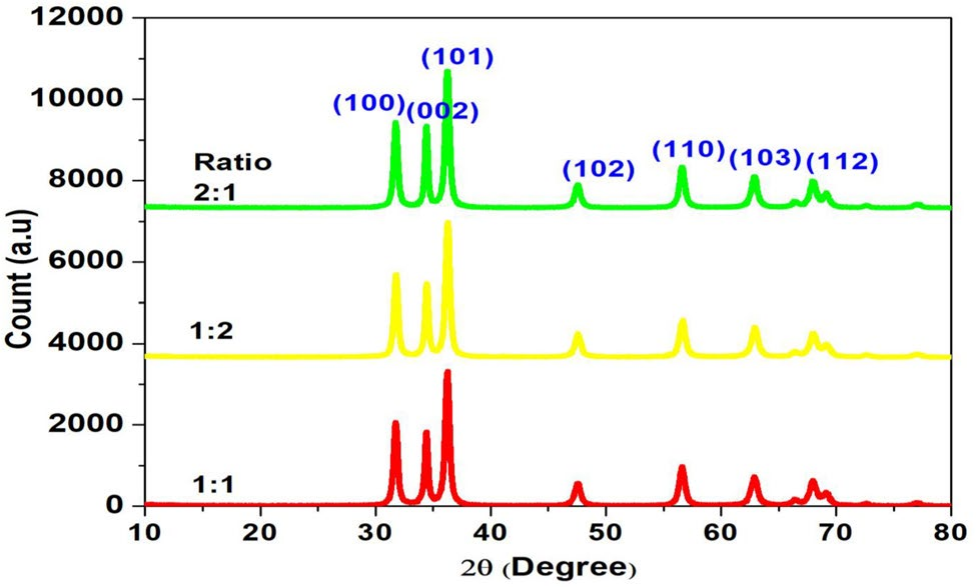
XRD spectra of ZnO NPs biosynthesized in 1: 1, 1: 2, and 2: 1 ratios of Zinc nitrate and fruit peel extract of *C. sinensis*

The formation of biosynthesized ZnO NPs was confirmed by X-ray diffraction measurements (Fig. 2). The X-Ray Diffraction (XRD) spectra depicted in figure (2) illustrated the crystalline structure of zinc oxide NPs (ZnO NPs) biosynthesized using different ratios of zinc nitrate to *C. sinensis* peel extract: 1:1, 1:2, and 2:1. The characteristic (major) peaks observed at specific 2θ angles correspond to crystal planes (1 0 0), (0 0 2), (1 0 1), (1 0 2), (1 1 0), (1 0 3), and (1 1 2), which are indicators of the hexagonal wurtzite structure of ZnO. All three samples exhibited these distinct peaks, confirming the successful synthesis of ZnO NPs with a consistent crystal phase across the different ratios. However, peak intensities varied among samples, with the 2:1 ratio sample exhibiting the highest intensities, particularly at the (1 0 1) plane, indicating superior crystallinity or larger crystallite size. In contrast, the 1:2 ratio sample showed lower peak intensities, which indicates comparatively reduced crystallinity or smaller NP size when a higher amount of *C. sinensis* peel extract is used relative to zinc nitrate.

The average crystallite size of the ZnO NPs was estimated to be 22.79, 23.01, and 24.21 nm for the 1:1, 1:2, and 2:1 precursor to extract ratios, respectively. The NPs synthesized at the 1:1 ratio exhibited the smallest crystallite size (22. 79 nm), while those synthesized at the 2:1 ratio showed a relatively larger crystallite size (24.21 nm). This trend is attributed to the higher relative volume of *C. sinensis* peel extract present in the 1:1 synthesis mixture. The increased concentration of bioactive phytochemicals in the extract provides a greater number of capping and stabilizing agents, which adsorb onto the surface of the nucleating ZnO crystals and inhibit their further growth, resulting in smaller crystallites [45, 46]. Conversely, at the 2:1 ratio, the lower relative volume of extract provides fewer capping molecules, leading to insufficient stabilization and allowing for continued crystal growth, thereby yielding slightly larger crystallites.

### FTIR analysis

**Fig. 3:**
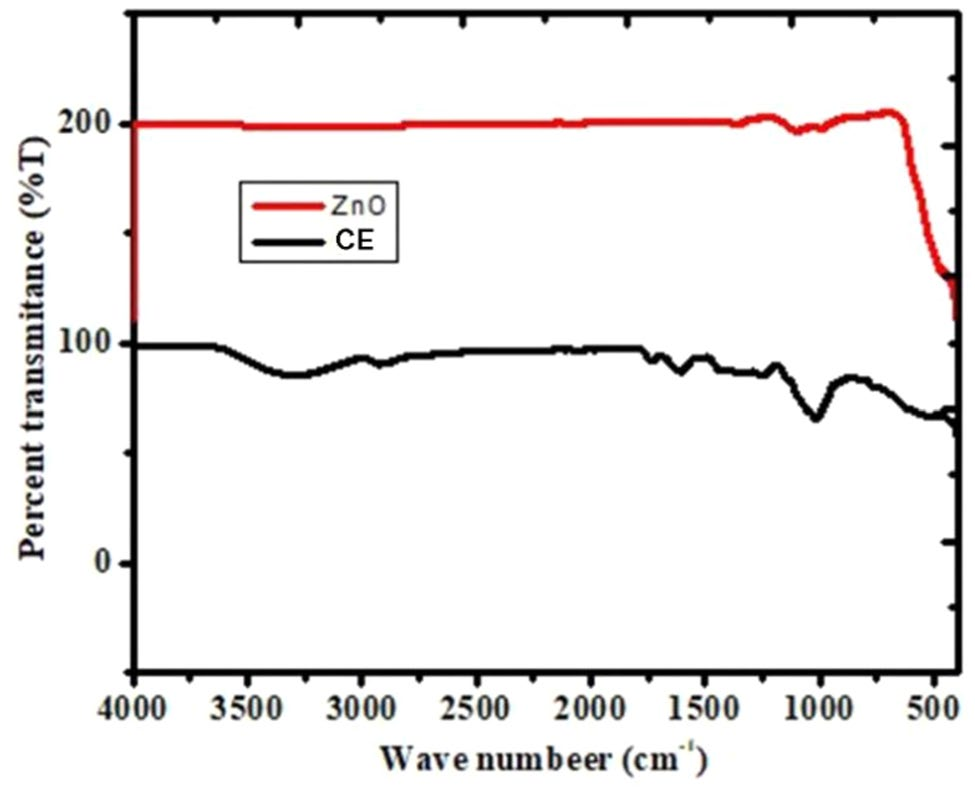
FTIR- spectra of ZnO and *C. sinensis* peel extract (CE)

The FTIR spectra presented compare the percent transmittance of ZnO and CE samples across a range of wave numbers from 4000 cm^− 1^ to 500 cm^− 1^ (Fig. 3). The ZnO sample illustrated by the red line maintained an exceptionally high transmittance (near or above 200%) across most of the spectrum, except for a significant drop below 600 cm^− 1^. This reduction is typical for Zn-O stretching vibrations, confirming the presence of zinc oxide NPs and suggesting the ZnO sample is pure and largely free from organic or moisture contaminants [47, 48, 49].

In contrast, the *C. sinensis* peel extract sample depicted by the black line demonstrated a more moderate transmittance (around 100%) with distinct absorption features, especially in the vibrational modes of various functional groups common in organic compounds, such as O-H, C-H, C = O, and C-O, indicating the complex molecular structure of the phytochemicals present in the extract [50, 51]. The transmittance of *C. sinensis* peel extract further decreases below 1000 cm^− 1^, highlighting enhanced molecular interactions and fingerprint region vibrations characteristic of polysaccharides, glyosidic bonds, and aromatic compounds [52].

The comparison clearly showed that ZnO is much more transparent in the infrared range due to its inorganic crystalline nature, while *C. sinensis* peel extract exhibits a diverse set of absorption bands resulting from its organic composition. The FTIR data thus effectively distinguished between the purity and inorganic character of ZnO and the functional complexity of *C. sinensis* peel extract, showing the importance of FTIR in nanomaterial characterization [52].

The most critical feature confirming the successful synthesis is the strong and broad absorption band observed below 600 cm^− 1^, centering around 500 cm^− 1^. This peak is assigned to the characteristic stretching vibration of the Zn-O bond in the tetrahedral coordination of the wurtzite crystal structure [47, 48]. The formation of this bond occurs during the calcination step, where the zinc hydroxide intermediate (formed by precipitation with NaOH) decomposes to form stable, crystalline zinc oxide. The broadening of this peak is typical for nanoparticles and correlates with the small crystallite size (22.79) observed in the XRD analysis [53].

**Table 1:** Fourier transform infrared (FTIR) peak assignments for biosynthesized ZnO nanoparticles

Wave number (cm^− 1^)	Transmittance (ZnO %)	Assignment (Functional group)
3400–3500	~ 95%	C = O stretching (Alcohols/Phenols/Ketones)
2920 − 2850	~ 99%	C-H Stretching (Alkyl groups)
1620–1640	~ 98%	C = O Stretching (Amides/Ketones)
1380–1450	~ 98%	C-O/C-H Bending (Carboxylate)
1050–1150	~ 90%	C-O stretching (polyphenols)
400–550	~ 80%	Zn-O Stretching (Metal-Oxygen bond)

As presented in Table 1, the peak at 3400 cm^− 1^ confirms the hydrophilic nature of the nanoparticles. The absence of strong organic peaks (C = O, C-H) between 2800 –1700 cm^− 1^ indicates that the *C. sinensis* peel extract served effectively as a capping agent during synthesis but was successfully removed during purification, leaving pure ZnO. The strong peak at 500 cm^− 1^ is definitive evidence of Zn-O bond formation.

### UV-vis spectroscopy analysis

**Fig. 4:**
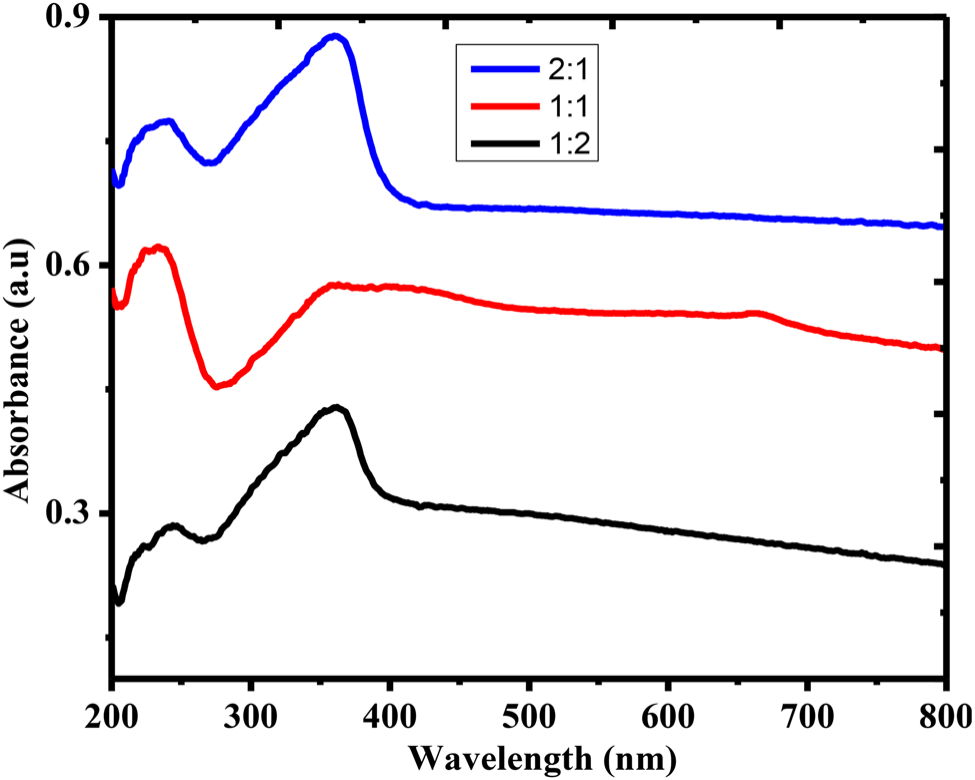
UV-Vis spectra of ZnO NPs

UV-vis spectra of ZnO NPs synthesized at different ratios (2:1, 1:1, and 1:2) exhibited distinctive absorbance profiles in the 200–800 nm wavelength range (Fig. 4). All samples demonstrate a prominent absorbance peak 350–380 nm region (Fig. 4), which is characteristic of the intrinsic bandgap absorption of ZnO NPs, corresponding to electron transmission from the valence band to the conduction band. The appearance of a peak in the UV-Vis spectra confirms the semiconducting nature, good crystallinity, and nanoscale dimensions of the biosynthesized ZnO NPs. The 2:1 ratio sample exhibited the highest absorbance intensity, followed by the 1:1 and 1:2 ratios, indicating higher NP concentration and better dispersion that enhanced light absorption [54]. Variations in peak intensity and slight shifts in spectral features across the different ratios suggest changes in NP size, degree of agglomeration, or surface characteristics influenced by the synthesis conditions [55].

The strong UV absorbance confirms the potential of these ZnO NPs for applications requiring UV light absorption, such as in UV-blocking materials, photo catalysts, and sensors. UV-vis analysis revealed that NP optical properties were significantly influenced by precursor ratios during synthesis. UV-vis analysis (Fig. 4) revealed 370 nm peaks diagnostic of pure ZnO phase, with 2:1 ratio elevated absorbance signaling enhanced yield, dispersity versus other ratios consistent with green synthesis studies [55, 56] linking formulation tuning to size modulated optics and synergistic antibacterial and photocatalytic potential, approaching ampicillin benchmarks eco-friendly.

### Scanning electron microscopy-energy dispersive spectroscopy (SEM-EDS) analysis

**Fig. 5:**
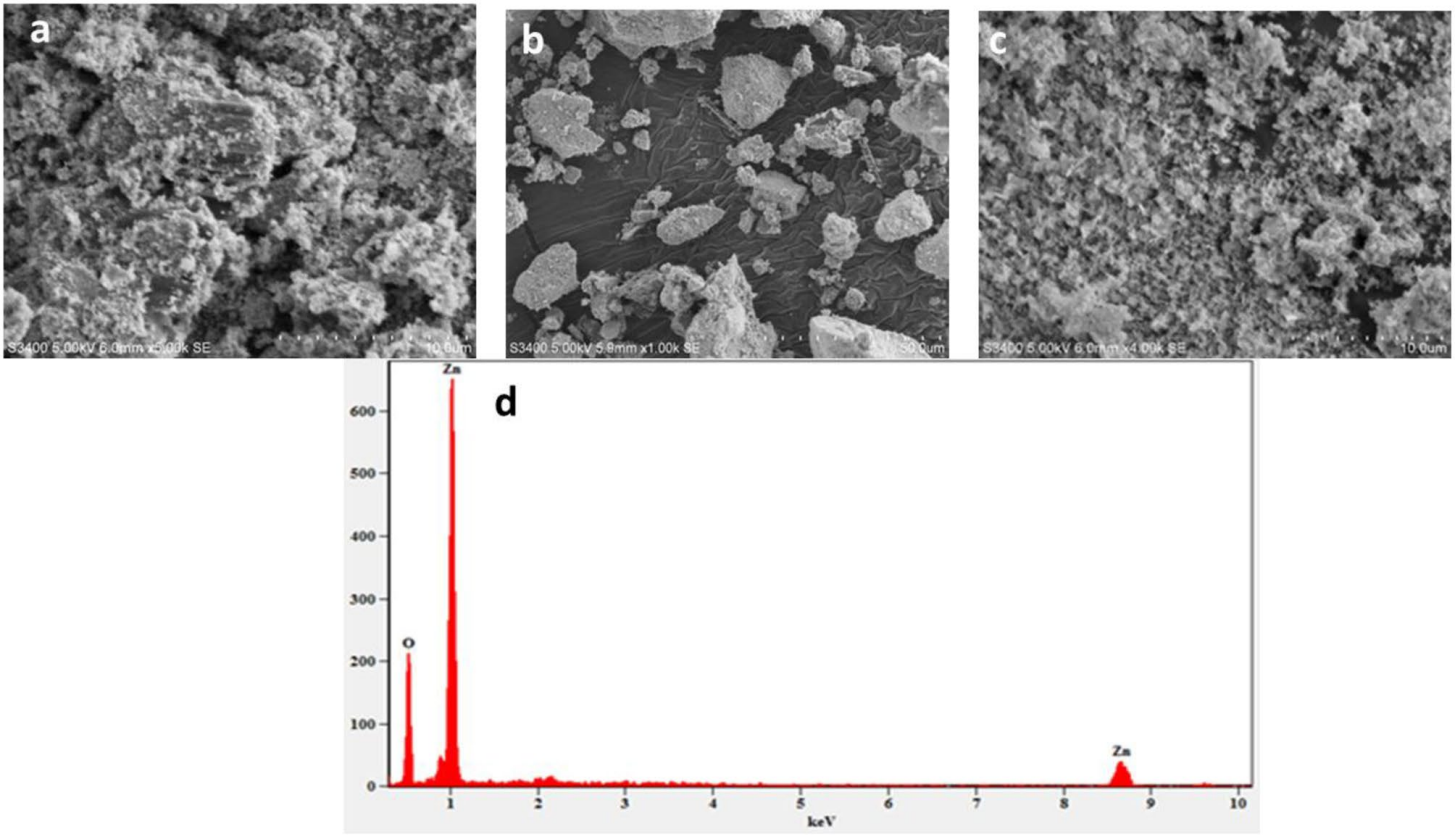
SEM image of ZnO: (**a**) 1:2, (**b**) 1:1, (**c**) 2:1 volume ratio, respectively) NPs and (**d**) EDS image of ZnO (1:1) NPs

The 1:1 ratio was selected as a representative sample for detail characterization, due to this ratio consistently exhibited the smallest crystallite size, enhancing surface reactivity, and optimal capping efficiency. SEM images of ZnO NPs synthesized at different volume ratios (1:2, 1:1, and 2:1) revealed significant morphological variations and aggregation differences. At the 1:2 volume ratio (Fig. 5a), ZnO NPs exhibited high aggregation, forming dense clusters with irregular, rough textures indicative of particle agglomeration. ZnO NPs at a 1:1 ratio (Fig. 5b) showed more uniform dispersion with reduced aggregation, displaying a narrower size distribution and more distinct particle boundaries. This suggested the improved control over particle formation and stabilization at this ratio. In contrast, the 2:1 volume ratio sample (Fig. 5c) showed a marked change in morphology with the NPs adopting more elongated rod-like structures. This anisotropic growth likely results from modified synthesis dynamics at higher volume ratios, which favors directional crystal growth. These morphological differences critically influenced ZnO NP functional properties, including surface area, catalytic activity, and optical behavior [58]. The SEM analysis demonstrated that adjusting the volume ratio during synthesis effectively controls the size, shape, and aggregation state of ZnO NPs. This provides valuable insight for tailoring ZnO NP properties to specific applications.

**Fig. 6:**
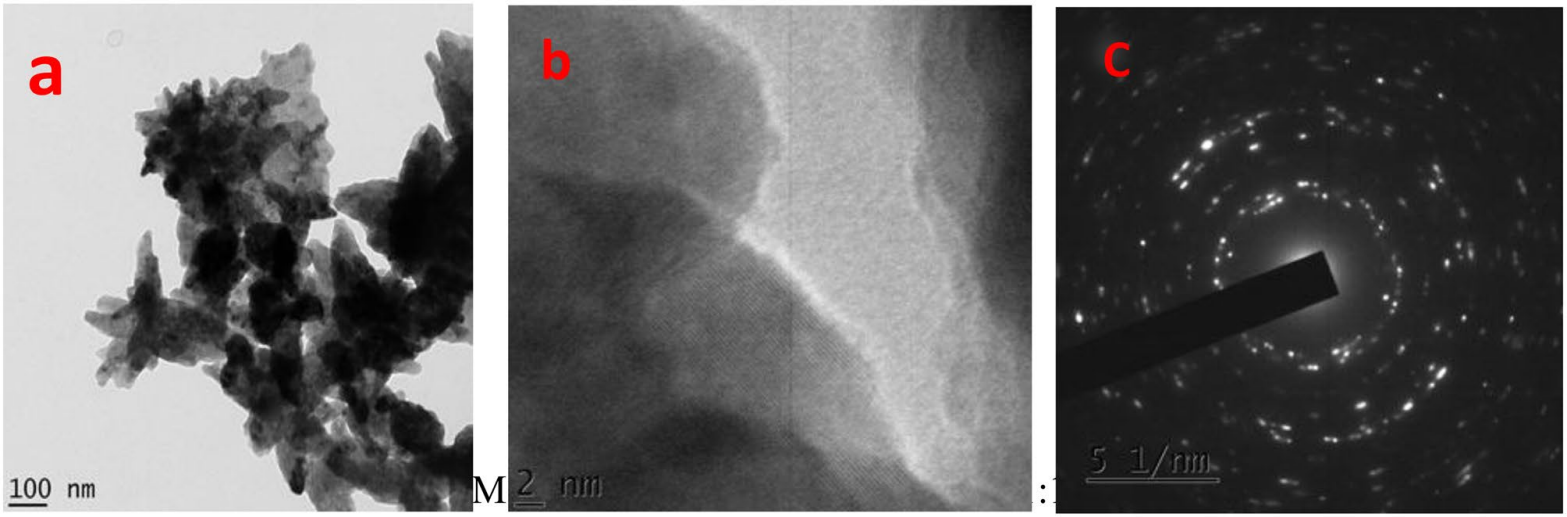
(**a**) TEM, (**b**) HRTEM, and (**c**) SAED pattern of ZnO (1:1) NPs

The EDS analysis of the biosynthesized ZnO NPs confirms the successful formation of zinc oxide with high purity (Fig. 6). The spectrum shows prominent peaks corresponding to zinc and oxygen elements, which are the key constituents of ZnO. The most intense peak at approximately 1 keV belongs to zinc, indicating its predominance in the sample, while the smaller peak nearby corresponds to oxygen. Additionally, a significant peak for zinc appears at around 8.60 keV. This verifies the presence of zinc in the NPs [59]. The absence of peaks from other elements suggested that the biosynthesis process yields pure ZnO NPs without contaminations. The strong signals for both zinc and oxygen elements confirm the oxidation state necessary for ZnO, supporting that the NPs maintain the expected stoichiometry [60].

### Transmission electron microscopy (TEM)

Building on the SEM observations, which revealed that the 1:1 precursor to extract ratio exhibited the uniform dispersion with reduced aggregation among the synthesized samples, TEM analysis was performed to further investigate the detailed morphology, particle size distribution, and crystallinity of this optimal formulation.

Figure 6 illustrates the morphological and structural features of ZnO NPs (1:1 ratio) observed via advanced electron microscopy. In the TEM image (Fig. 6a), the NPs appear as aggregated clusters with irregular shapes and sizes, most of which are well below 100 nm, indicating successful nanostructure formation [60, 61]. The TEM micrograph of ZnO NPs synthesized at the 1:1 ratio exhibited a predominantly spherical to hexagonal morphology with relatively smooth surfaces. Importantly, the TEM findings, showing well-dispersed NPs with minimal agglomeration. This uniform dispersion is attributed to the effective capping action of phytochemicals present in the *C. sinensis* peel extract, which prevented excessive particle growth and aggregation during synthesis.

The high-resolution TEM image (Fig. 6b) further reveals the fine crystalline structure of these particles, displaying clear measurements of the lattice fringes (0.26 nm) that confirm their highly ordered and crystalline nature. This structural order at the atomic scale demonstrates the material purity and suggests that the synthesis technique produced ZnO NPs with high crystallinity [63]. Complementing these observations, the SAED pattern (Fig. 6c) shows concentric diffraction rings and distinct spots characteristic of polycrystalline ZnO NPs with pronounced crystal domains. The sharp rings and spots confirm that the ZnO sample comprises multiple crystalline grains, likely oriented but retaining the wurtzite structure typical of ZnO [59]. Collectively, the TEM analysis confirmed well-defined, polycrystalline ZnO NPs with highly ordered internal structure, suitable for technological applications.

### Antibacterial activity

The antibacterial activity of ZnO NPs synthesized at different precursor to extract ratios (1:1, 1:2, and 2:1) was evaluated against two Gram negative bacteria (*Escherichia coli* and *P. aeruginosa)* and two Gram positive bacteria (*Staphylococcus aureus* and *Streptococcus pyogenes*) using the agar well diffusion method. The zone of inhibition was measured and are presented separately for each bacterial strain in Table 2, as different bacterial species exhibit varying susceptibilities to both NPs and antibiotics.

All ZnO NPs formulations exhibited concentration dependent antibacterial activity against all tested strains, with larger inhibition zones observed at higher concentrations (100 µg/mL). The 2:1 ratio consistently showed the largest inhibition zone across all bacterial strains, followed by the 1:2 and 1:1 ratios. Against *S. aureus*, the 2:1 ratio at 100 µg/mL produced an inhibition zone of 18 mm, approaching the positive control ampicillin (20 mm). Against *P. auruginosa*, the 2:1 ratio at 100 µg/mL showed 16 mm inhibition compared to 19 mm of ampicillin.

Gram- positive bacteria (*S. aureus* and *S. pyogenes*) were generally more susceptible to ZnO NPs than Gram- negative bacteria (*E. coli* and *P. auruginosa*). The diference can be attributed to variations in cell well structure; the thick peptidoglycan layer of Gram-positive bacteria my facilitate NPs interaction, while the outer membrane of Gram-negative bacteria acts as an additional permeability barrier [53].

These findings align closely with prior literature, where chemically synthesized or sol-gel ZnO NPs reported zone of inhibition 13.50–24 mm against similar pathogens [56, 58, 62], while green synthesized variants ranged 10–17 mm, positioning the 2:1 ratio as comparably effective to advanced methods without matching ampicillin benchmark of 17.00 ± 2.8 0 mm [64].

**Table 2:** Antibacterial activity **(**zone of inhibition) of ZnO NP formulations and ampicillin against four bacterial strains (*Escherichia coli*,* Staphylococcus aureus*,* P. aeruginosa* and *Streptococcus pyogenes*)

Precursor salt: plant extract ratio	Concentration (µg/mL)	E. Coli (mm)	*P*. aeruginosa (mm)	S. aureus (mm)	S. pyogenes (mm)
ZnO (1:1)	50	7	8	8	7
	75	8	10	10	7
	100	10	12	12	8
ZnO (1:2)	50	8	11	9	7
	75	9	12	11	7
	100	10	14	14	8
ZnO (2:1)	50	10	12	11	8
	75	12	14	16	9
	100	13	16	18	11
Ampicillin	100	14	19	20	15

**Fig. 7:**
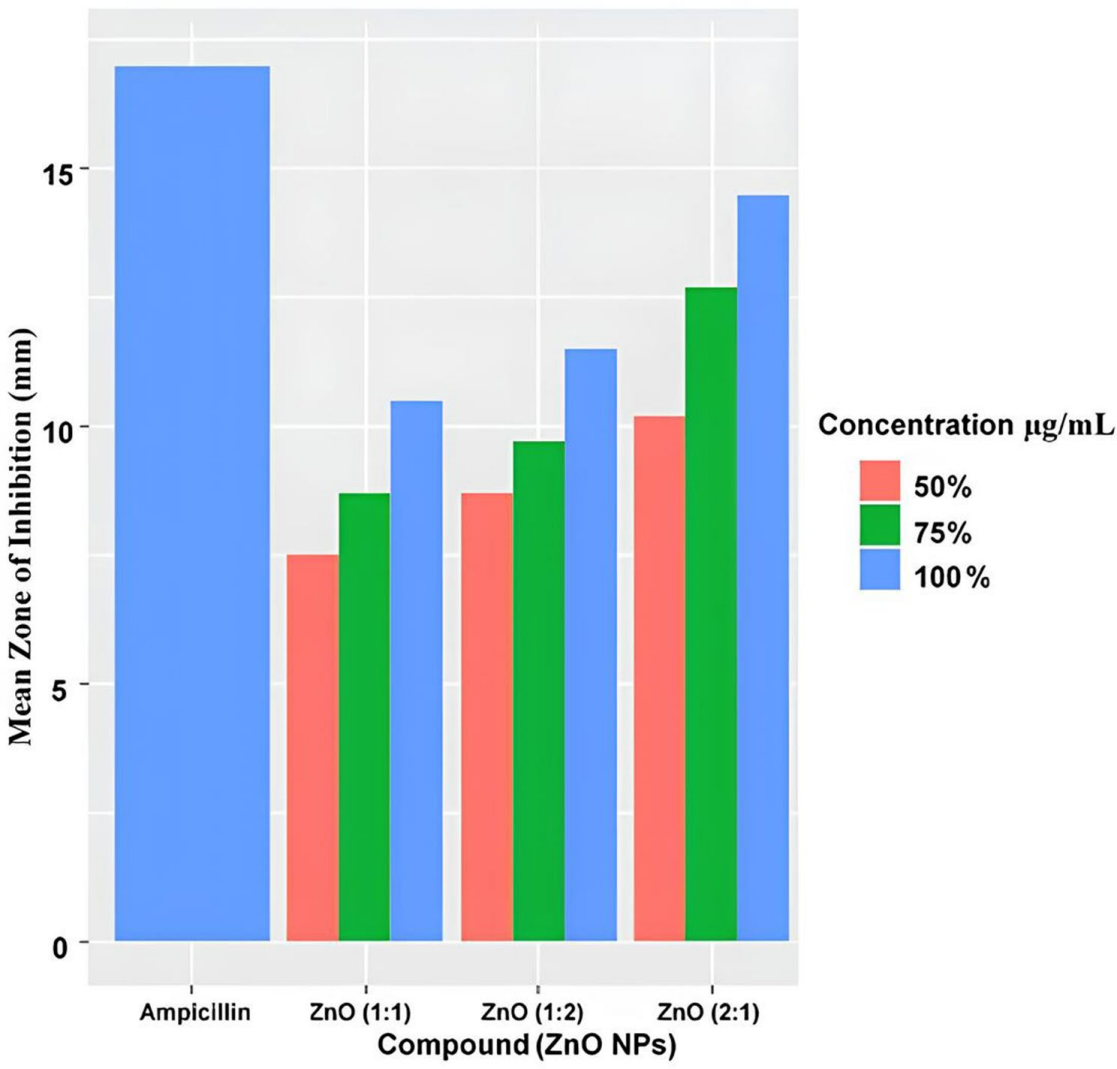
Antibacterial zone of inhibition by ZnO NPs at varying concentrations

Figure 7 illustrates the mean zone of inhibition (mm) for grean synthesized ZnO nanoparticles (ZnO 1:1, 1:2, and 2:1 ratios) at dofferent concentrations against a bacterial pathogen, compared to Ampicillin standard, at 50%, 75%, and 100% concentrations. ZnO (2:1) ratio at 100% concentration achieves the largest inhibition zone, surpassing Ampicillin and all other treatments. ZnO (1:2) ratio shows moderate antibacterial activity, while lower concentration (50%) yield smaller zone of inhibition across all (7–11 mm). 75% concentration indicates consistent dose-response trends, with higher concentrations enhancing efficacy through increased ROS and Zn^2+^ release.The antibacterial activity of ZnO NP was dose-dependent and the finding align with previous studies on biosynthesized ZnO NPs [64, 65].

**Fig. 8:**
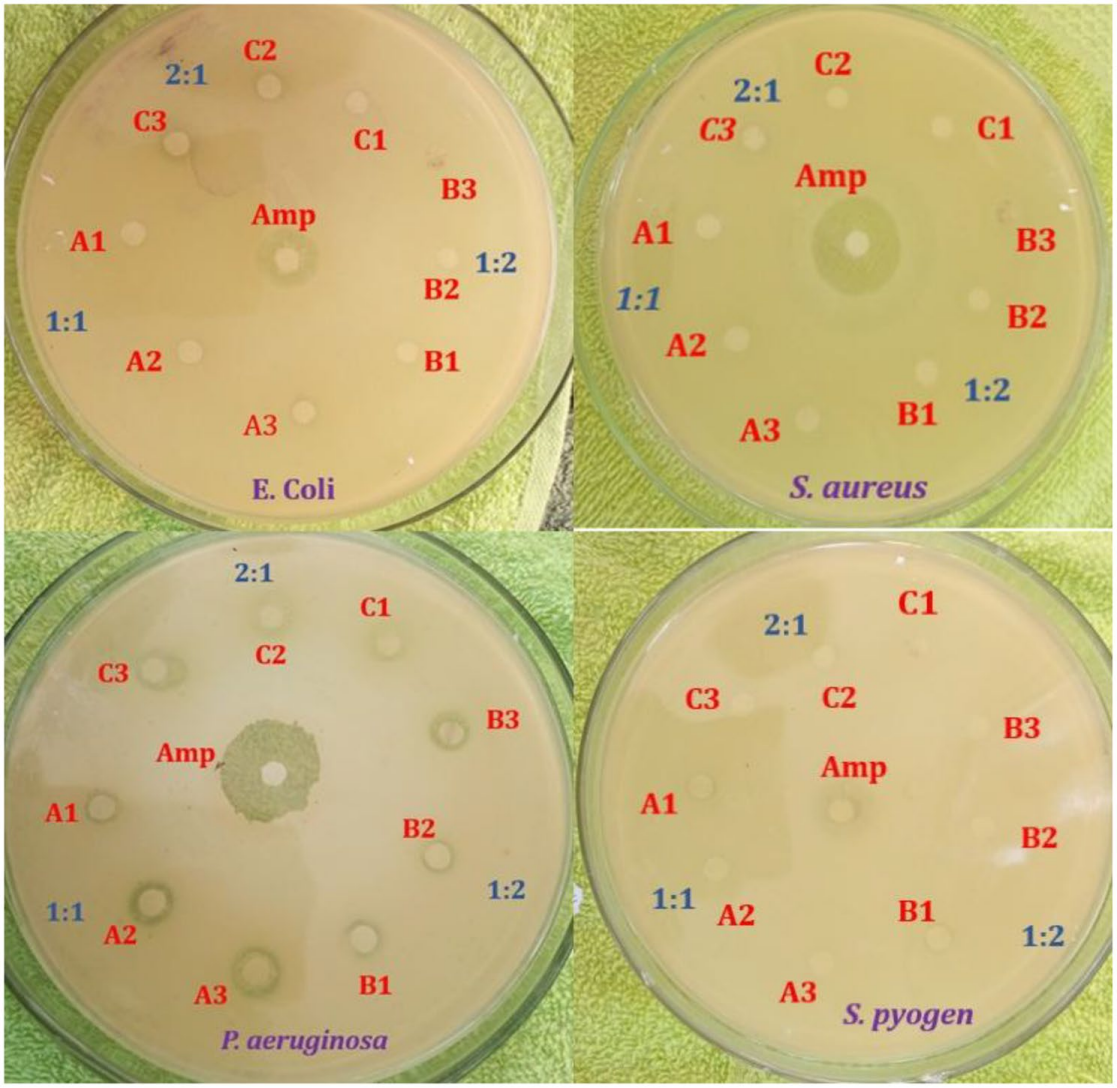
Antibacterial effect of ZnO NPs against *E. Coli*,* S. aureus*,* P. aeruginosa* and *S. pyogen*

Figure 8 illustrates the antibacterial efficacy of ZnO NPs against four clinically relevant bacterial strains evaluated via the Kirby-Bauer disk diffusion assay on Mueller-Hinton agar. Each Petri dish displays zones of inhibition around disks loaded with test samples (A1-A3, B1-B3, C1-C3, likely representing varying NP concentrations of formulations) and ampicillin (Amp) as the positive control, with replicates denoted as 1:1, 2:1, and 1:2. Prominent inhibition zones were observed for *E. coli* around Amp, A1, B1-B2, and C2, indicating strong susceptibility, while S. aureus showed large zones primarily with Amp and C1, alongside moderate effects from A1, B2, and C3. In contrast, *P. aeruginosa* exhibited minimal inhibition across most samples, including Amp, consistent with its intrinsic resistance mechanisms such as efflux pumps and outer membrane barriers. These concentration dependent zone of inhibition (Table, Figs. 7 and 8) confirming prior reports on green ZnO NPs [56, 57, 66].

### Preservative activity

Table 3 demonstrates that ZnO NP coatings at ratios of 1:1, 1:2, and 2:1 with concentrations of 50%, 75%, and 100% (w/v) significantly reduce weight loss in banana samples over 14 days of storage, ranging from 21% to 26.70% compared to 47.70% in the water-dipped (negative control). The average daily weight loss for coated samples varied from 1.90 to 3.00 g/day, evenly half of that of the control (5.0 g/day), with the lowest losses observed at ZnO (1:2) 75–100% (21-21.30%) and ZnO (2:1) 75% (21.40%), indicating optimal NP dispersion and barrier properties that limit transpiration and microbial respiration.

**Table 3:** Weight loss of banana samples coated with ZnO NPs over 14 days of storage

Compound (Coating Material)	Concentration (w/v)	Initial (g)	Final (g)	% loss	Avg. Daily loss (g/day)
ZnO (1:1)	50%	159.20	120.00	24.60	2.80
	75%	152.70	118.00	22.70	2.50
	100%	158.20	115.90	26.70	3.00
ZnO (1:2)	50%	146.60	109.00	25.60	2.70
	75%	154.50	122.00	21.00	2.30
	100%	141.10	111.00	21.30	2.10
ZnO (2:1)	50%	134.80	102.00	24.30	2.30
	75%	126.60	99.50	21.40	1.90
	100%	151.80	115.70	23.80	2.60
Water (-ve control)	100%	149.10	78.00	47.70	5.00

**Fig. 9:**
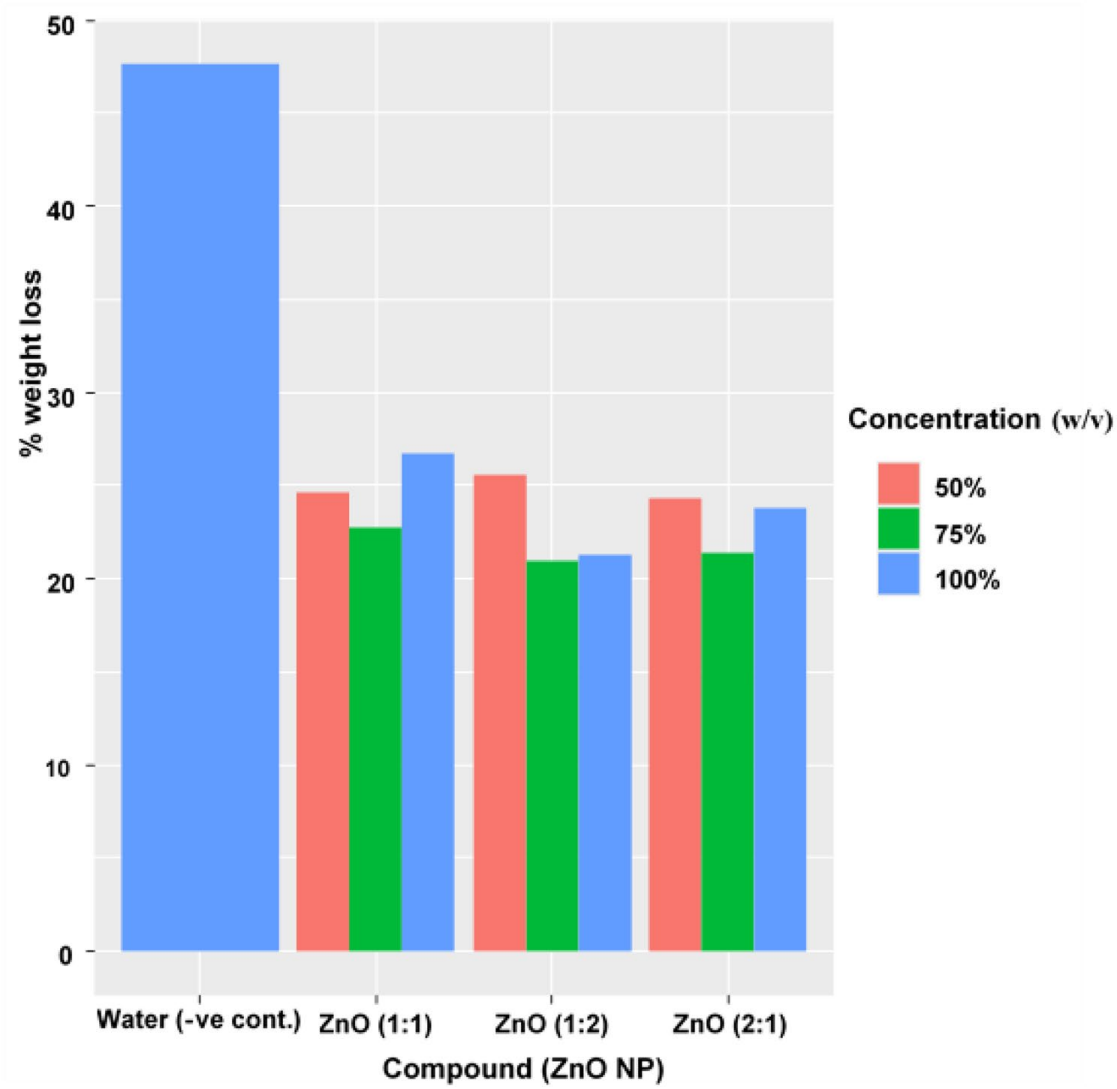
Weight loss (%) of Banana samples coated with ZnO NPs after 14 days of storage

The bar graph (Fig. 9) illustrates that banana samples coated with ZnO NPs at increasing concentrations (50%, 75%, and 100%) exhibited significantly reduced weight loss after 14 days of storage compared to water control, with losses dropping from approximately 50% in the control to around 10% at 100% ZnO NP concentration. This trend underscores the efficacy of ZnO NP coatings in forming a protective barrier that minimizes transpiration, respiration, and microbial-induced moisture loss, thereby extending postharvest shelf life. Similarly, Aruwajoye et al. [67] reported that ZnO/Ag_2_O nanoparticle treatments on bananas reduced decay incidence to 44.44% versus 100% in controls, while maintaining lower weight loss through modulated ripening and turgor preservation. In comparable fruit studies, Leta et al. [68] observed pomegranate arils coated with banana powder/cellulose nanofiber/ZnO NPs achieving only 0.44–0.90% weight loss after 18 days at cold storage, attributing this to enhanced gas barrier properties and reduced metabolic activities: sugar consumption and cell wall degradation. Anean et al. [69] further corroborated these findings in pomegranates, where chitosan coatings loaded with 0.06% ZnO NPs limited weight loss and microbial growth for up to 20 days.

These finding align with the broader literature on nano-edible coatings, where ZnO NPs at concentrations of 0.06-3% integrated into chitosan or starch matrices have consistently lowered weight loss in fruit such as strawberries and salak by inhibiting evaporation, pathogen proliferation, and oxidative process. For instance, Sani et al. [70] demonstrated that 3% ZnO NP-chitosan coatings on strawberries preserved mass integrity over extended storage, mirroring the concentration dependent protection observed. La et al. [71] also noted maintained banana firmness and reduced weight loss with ZnO NP coatings, emphasizing their role in sustaining physicochemical quality parameters. The observed dose response effect in this study optimal at 100% ZnO NP suggests an ideal formulation threshold for industrial application, potentially reducing postharvest losses in banana supply chains, which account for significant global food waste; however, future research should evaluate sensory attributes, nanoparticle migration and long term biosafety to ensure commercial viability.

## Conclusion

The biological synthesis of zinc oxide NPs using *C. sinensis* peel extract demonstrated a clear dependence on the volume ratio of zinc nitrate to extract, significantly influencing their structural, morphological, and functional properties. X-ray diffraction (XRD) analysis confirmed the successful synthesis of crystalline ZnO NPs with a hexagonal wurtzite structure across all ratios, and the 2:1 ratio exhibited superior crystallinity and larger crystallite size. Complementary analysis, including FTIR, UV-vis, SEM-EDS, and TEM, reinforced the purity, optical properties, and well-defined crystalline morphology of the NPs, which showed that increasing zinc nitrate relative to extract favors enhanced crystallinity and modified particle shapes, such as elongated rods. Functionally, the 2:1 ZnO NPs exhibited the most potent antibacterial activity against tested bacterial strains and provided the best preservative effect in weight retention studies of nano-coated bananas. Based on the comprehensive characterization and application testing, the **2:1 precursor-to-extract ratio** (zinc nitrate : *C. sinensis* peel extract) was identified as the optimal formulation. While the 1:1 ratio produced the smallest crystallite size (22.79 nm), the 2:1 ratio demonstrated superior performance in the key application assays that are the focus of this study. This study highlights the critical role of precursor ratios in tailoring ZnO NP characteristics and validates their promising potential as effective antibacterial and preservative agents for biomedical and food preservation applications.

## Data Availability

All data generated or analyzed during this study are included in the manuscript.
